# PpERF3 positively regulates ABA biosynthesis by activating *PpNCED2/3* transcription during fruit ripening in peach

**DOI:** 10.1038/s41438-018-0094-2

**Published:** 2019-02-01

**Authors:** Xiaobei Wang, Wenfang Zeng, Yifeng Ding, Yan Wang, Liang Niu, Jia-Long Yao, Lei Pan, Zhenhua Lu, Guochao Cui, Guohuai Li, Zhiqiang Wang

**Affiliations:** 1 Zhengzhou Fruit Research Institute, Chinese Academy of Agricultural Sciences, 450009 Zhengzhou, China; 2 Key Laboratory of Horticultural Plant Biology, Ministry of Education, Huazhong Agricultural University, 430070 Wuhan, China; 3 The New Zealand Institute for Plant & Food Research Limited, Auckland, New Zealand

**Keywords:** Plant signalling, Transcriptional regulatory elements

## Abstract

The plant hormone ethylene regulates ripening in climacteric fruits. The phytohormone abscisic acid (ABA) affects ethylene biosynthesis, but whether ethylene influences ABA biosynthesis is unknown. To explore this possibility, we investigated the interactions between the ABA biosynthesis genes *PpNCED2/3* and the ethylene response transcription factor PpERF3 in peach fruit. The ABA content increased during fruit maturation and reached a peak at stage S4 III. The increase was greatly inhibited by the ethylene inhibitor 1-MCP, which also suppressed *PpERF3* expression. *PpERF3* shared a similar expression profile with *PpNCED2/3*, encoding a rate-limiting enzyme involved in ABA biosynthesis, during fruit ripening. A yeast one-hybrid assay suggested that the nuclear-localized PpERF3 might bind to the promoters of *PpNCED2/3*. PpERF3 increased the expression of *PpNCED2/3* as shown by dual-luciferase reporters, promoter-GUS assays and transient expression analyses in peach fruit. Collectively, these results suggest that ethylene promotes ABA biosynthesis through PpERF3’s regulation of the expression of ABA biosynthesis genes *PpNCED2/3*.

## Fruit ripening: Hormones go hand in hand

Two hormones that regulate fruit ripening are more closely linked than previously thought, according to a study of ripening in peaches. Ethylene is a key ripening hormone in many fruits, and high ethylene levels turn on ethylene response factors (ERFs), genes that trigger production of sugars, pigments, and flavor compounds associated with ripening. Another hormone, abscisic acid (ABA), has recently been found to affect ripening, but its interaction with ethylene is unclear. Zhiqiang Wang and Guohuai Li at the Chinese Academy of Agricultural Sciences and coworkers investigated how ethylene and ABA interact during ripening. They found that as ethylene levels increased, ABA production was stimulated. Further investigation showed that ethylene directly triggered the ABA increase via ERFs. These results illuminate the fruit ripening process, and may help in finding ways to prolong fruit shelf life.

## Introduction

Ethylene is the key plant hormone that regulates the fruit ripening process. This regulation is mediated through ethylene-responsive genes, particularly those encoding transcription factors. These transcription factors regulate multiple biochemical pathways that underpin fruit ripening traits, including the levels of sugars, acids, pigments, and flavor and aroma compounds and fruit firmness and texture^1,2^. In the past few decades, our understanding of the mechanisms by which plants respond to ethylene has greatly increased^3,4^. Ethylene biosynthesis is catalyzed by two key enzymes: ACC synthase and ACC oxidase. The expression of the genes encoding these two ethylene enzymes is modulated by ethylene-responsive transcription factors, thereby forming a feedback loop. Depending whether the transcription factor acts as a repressor or activator of transcription, the feedback loop may be autoinhibitory or autostimulatory. The autoinhibitory loop, also known as system 1 ethylene production, functions during the early stage of fruit development and is responsible for producing basal levels of ethylene. The autostimulatory loop, also known as system 2 ethylene production, functions during climacteric fruit ripening, and is responsible for producing high levels of ethylene^5^.

Abscisic acid (ABA) is another plant hormone that has regulatory roles during fruit development and ripening^6^, although it also controls plant wilting and stomatal closure^7^. ABA biosynthesis requires the cleavage of C40 carotenoids by 9-*cis*-epoxycarotenoid dehydrogenase (NCED) to form its direct precursor, xanthoxin. This cleavage process is a key rate-limiting step in ABA biosynthesis^8^. ABA levels sharply increase preceding the release of ethylene. The process of fruit ripening can be induced by the application of exogenous ABA^9^. Transgenic tomato (*Solanum lycopersicum*) fruits containing the *SlNCED1-RNAi* construct (to silence *SlNCED1*) exhibited inhibited cell wall degradation due to reduced levels of endogenous ABA, indicating that ABA has crucial roles in fruit ripening^10,11^. Many studies suggest that ABA affects ethylene production, likely by regulating the levels of ACC synthase (ACS) and ACC oxidase (ACO)^12,13^. However, little is known about whether ethylene affects ABA biosynthesis.

Ethylene response factors (ERFs) are plant-specific transcription factors. Many of them can mediate the transcription of ethylene-dependent genes^3,14,15^. Because ERFs comprise a family of transcription factors, different members of the family allow diverse and specific ethylene responses to occur in different plants^16^. ERF proteins can bind to DNA *cis*-acting elements such as a DRE (CCGAC), a GCC box (AGCCGCC), (A/G)CC(G/C)AC, and AA(T)TTCAAA motifs through its conserved ERF domain. These elements are found in the promoters of many ethylene-responsive genes, confirming that ERFs are involved in ethylene signaling or biosynthesis^3,4,16,17^. For example, tomato LeERF interacts with the GCC box and activates the expression of ethylene biosynthesis genes^18^. In banana (*Musa acuminata*), MaERF11 represses the expression of *MaACO1* and expansin genes via recruiting the histone deacetylase MaHDA1^4^. Apple (*Malus domestica*) MdERF2 interacts with the DRE motif in the promoter of the *MdACS1* gene and suppresses its transcription, thereby inhibiting ethylene biosynthesis in ripening fruit^3^. Although it well known that ERFs regulate fruit ripening through ethylene^1^, it is unclear whether they also control fruit ripening by the transcriptional regulation of ABA biosynthesis genes.

The peach (*Prunus persica*) gene *PpERF3* (Prupe.7G194400) shares similar expression patterns with *PpACS1* and is regulated by 1-MCP^19^, but the target genes of PpERF3 are unknown. In the present study, we found that PpERF3 directly bound to the promoters of *PpNCED* genes and enhanced their transcription. We also found that *PpNCED* promoter activity is positively regulated by ethylene. Our results show that ERFs regulate ABA biosynthesis in ripening peach fruit by targeting *PpNCED* promoters.

## Results

### ABA levels and *PpNCED2/3* expression during peach fruit ripening

**Fig. 1:**
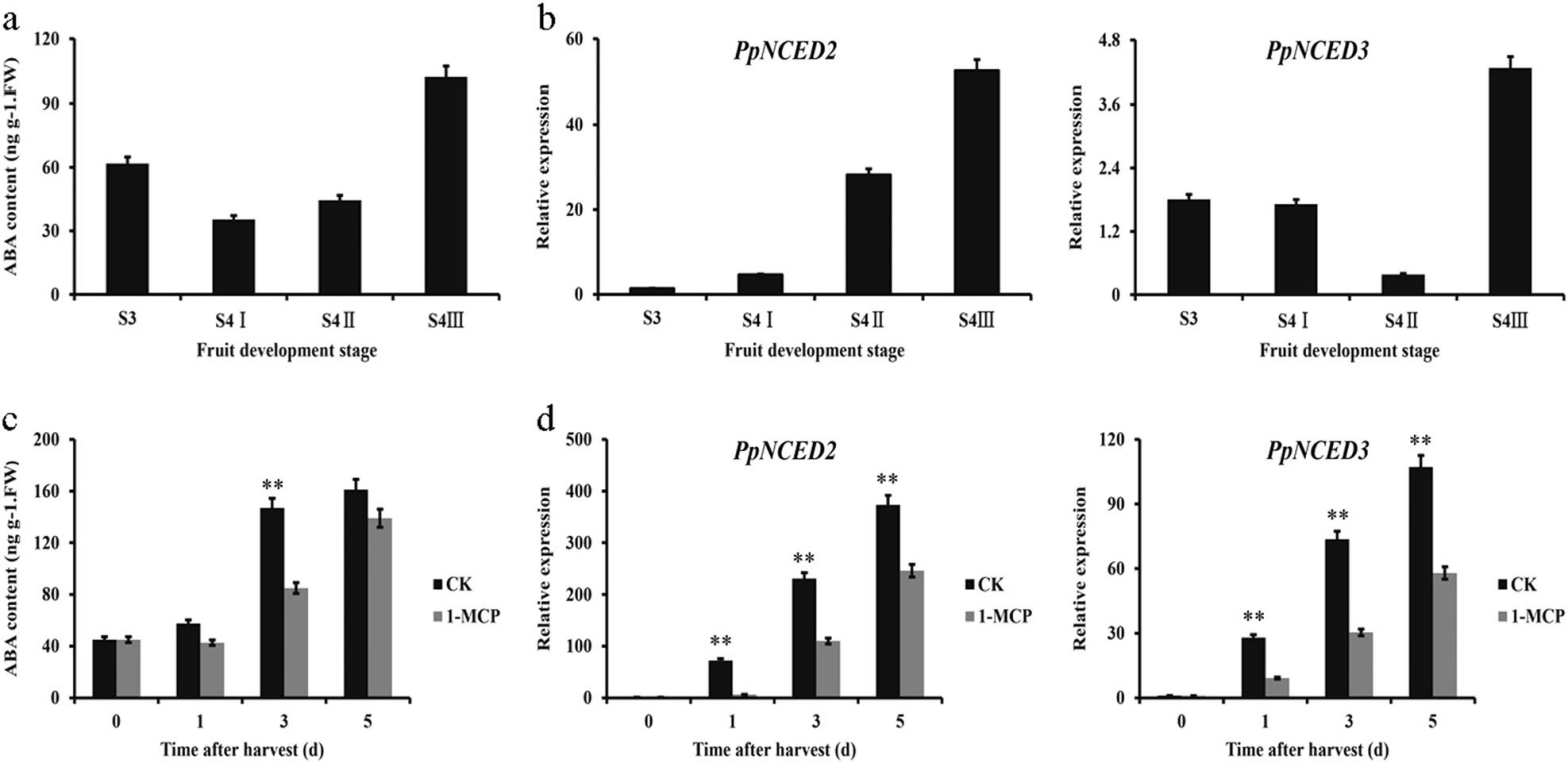
The effects of 1-MCP on ABA levels and *PpNCED2/3* expression in peach fruit. **a** ABA levels in peach fruit. **b** Expression levels of *PpNCED2/3* during peach fruit ripening. **c** ABA contents in fruit harvested at stage S4 II and treated with 0 or 10 µL L^−1^ of 1-MCP. **d** The expression levels of *PpNCED2/3* in peach fruit treated as in **c**. As a control (CK) treatment, 0 µL L^−1^ of 1-MCP was used. Values are means ± SD of three biological replicates

During the maturation of ‘CN13’ peach fruit, the ABA content decreased slowly from S3 to S4 I, which was followed by a gradual increase from S4 I to S4 II and a marked increase from S4 II to S4 III (Fig. 1a). Since PpNCED functions in a rate-limiting step in ABA biosynthesis, we analyzed the transcript profiles of *PpNCED* genes using transcriptome data and verified the results by qRT-PCR. Three *NCED* genes were found in peach, Prupe.1G061300, Prupe.4G082000, Prupe.4G150100, which were named *PpNCED1*, *PpNCED2*, and *PpNCED3*, respectively. The mRNA transcript level of *PpNCED2* remained low at stage S3 and then increased sharply from S4 I to S4 III. The mRNA transcript level of *PpNCED3* decreased at stage S4 II and then increased markedly at stage S4 III (Fig. 1b). In contrast, the *PpNCED1* transcript level remained low throughout fruit ripening (Supplementary Table S1).

We analyzed the correlation between ABA content and *PpNCED2/3* expression level in peach fruits subjected to 1-MCP treatment. We treated ‘CN13’ peach fruit harvested at stage S4 II with 1-MCP (0, 10 µL L^−1^) at 20 °C for 1 d to delay fruit ripening. ABA content was lower in fruit treated with 10 µL L^−1^ 1-MCP than in control fruit receiving 0 µL L^−1^ on days 1, 3, and 5, and a significant difference in ABA content was detected on day 3 (Fig. 1c). To identify the possible roles of *PpNCED2/3* in ABA biosynthesis, we analyzed their expression profiles in peach fruit after treatment with 1-MCP. Consistent with the above results, the expression levels of *PpNCED2/3* were strongly suppressed in ‘CN13’ fruit under 1-MCP treatment on days 1, 3, and 5 (Fig. 1d).

### Promoter elements identified using bioinformatics analysis

The *cis*-elements in the promoter sequences (i.e., in the 2, 2.9 kb region upstream of the translation start site of *PpNCED2* and *PpNCED3* genes, respectively) involved in plant hormone (especially ethylene) responses were identified using PlantCARE and a manual search to understand the transcriptional regulation of the *PpNCED2/3* genes. ERF (ethylene response factor) binding site, MeJA-responsive element, and abscisic acid-responsive element were found in the promoter of *PpNCED2*. These elements were also found in the promoter of *PpNCED3*, in addition to auxin-responsive element, gibberellin-responsive element, and MYB binding site (Table 1).

**Table 1:** *Cis*-acting regulatory elements were predicted in the promoter regions of *PpNCED2/3* related to fruit development and ripening in peach

Genes	*cis*-element^Z^	Sequence	Probable function
*PpNCED2*	CRT/DRE	A/GCCGAC	ERF (ethylene response factor) binding site
	CGTCA-motif	CGTCA	MeJA-responsive element
	TGACG-motif	TGACG	MeJA-responsive element
		CACGTG	
		TACGTG	
	ABRE	GCAACGTGTC	Abscisic acid-responsive element
		GCCACGTACA	
		ACGTGGC	
		ACGTGGC	
*PpNCED3*	ERE	ATTTCAAA	ERF (ethylene response factor) binding site
	CRT/DRE	A/GCCGAC	ERF (ethylene response factor) binding site
	TGA-element	AACGAC	Auxin-responsive element
	P-box	CCTTTTG	Gibberellin-responsive element
	TATC-box	TATCCCA	Gibberellin-responsive element
	GARE-motif	TCTGTTG	Gibberellin-responsive element
	ABRE	T/CACGTG	Abscisic acid-responsive element
	CGTCA-motif	CGTCA	MeJA-responsive element
	TGACG-motif	TGACG	MeJA-responsive element
	MBS	CGGTCA	MYB binding site
	MBSI	aaaAaaC(G/C)GTTA	MYB binding site

### *PpNCED2/3* promoter activity assays

**Fig. 2:**
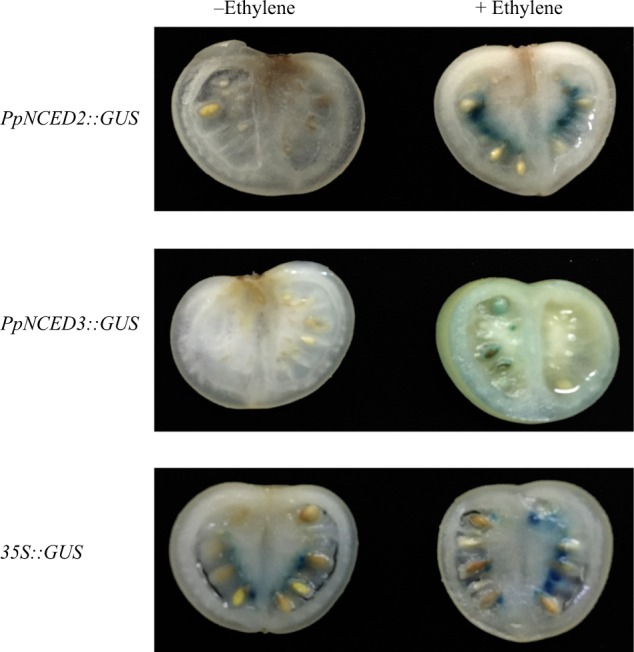
Analysis of the activity of *PpNCED2/3* promoters in tomato fruit. Tomato fruit at the breaker stage was infiltrated with *Agrobacterium* cells containing either *PpNCED2/3::GUS* or *35S::GUS* construct. At 3 days after infiltration, the fruit was sliced and tested for GUS activity using a GUS-staining solution with or without 10 mM ethylene

To further demonstrate that *PpNCED2/3* expression levels were enhanced by ethylene, we fused the *PpNCED2/3* promoters with the *GUS* reporter gene and transiently expressed these genes in tomato fruits. After 3 days, we treated the transiently transformed tomato fruits with ethylene and used untreated fruits as controls. As expected, *GUS* driven by the *PpNCED2/3* promoter was highly expressed in fruits treated with ethylene and not strongly expressed in untreated fruits. By contrast, *CaMV35Spro::GUS* was ubiquitously expressed in fruits regardless of ethylene treatment (Fig. 2). These observations suggested that *PpNCED2/3* expression is affected by ethylene.

### Phylogenetic tree analysis of PpERF3 and other ERFs

ERFs comprise one of the largest families of plant transcription factors. Phylogenetic analysis of PpERF3 and ERFs from other fruits, including tomato (*Solanum lycopersicum*), banana (*Musa acuminata*), kiwifruit (*Actinidia deliciosa*), melon (*Cucumis melo*), and apple (*Malus domestica*), revealed that PpERF3 clustered with MaERF9 from banana (Supplementary Fig. S1a). PpERF3 aligned with many functionally characterized AP2/ERF-type proteins from various plant species, revealing high homology within the AP2/ERF domain (Supplementary Fig. S1b).

### PpERF3 is localized to the nucleus

**Fig. 3:**
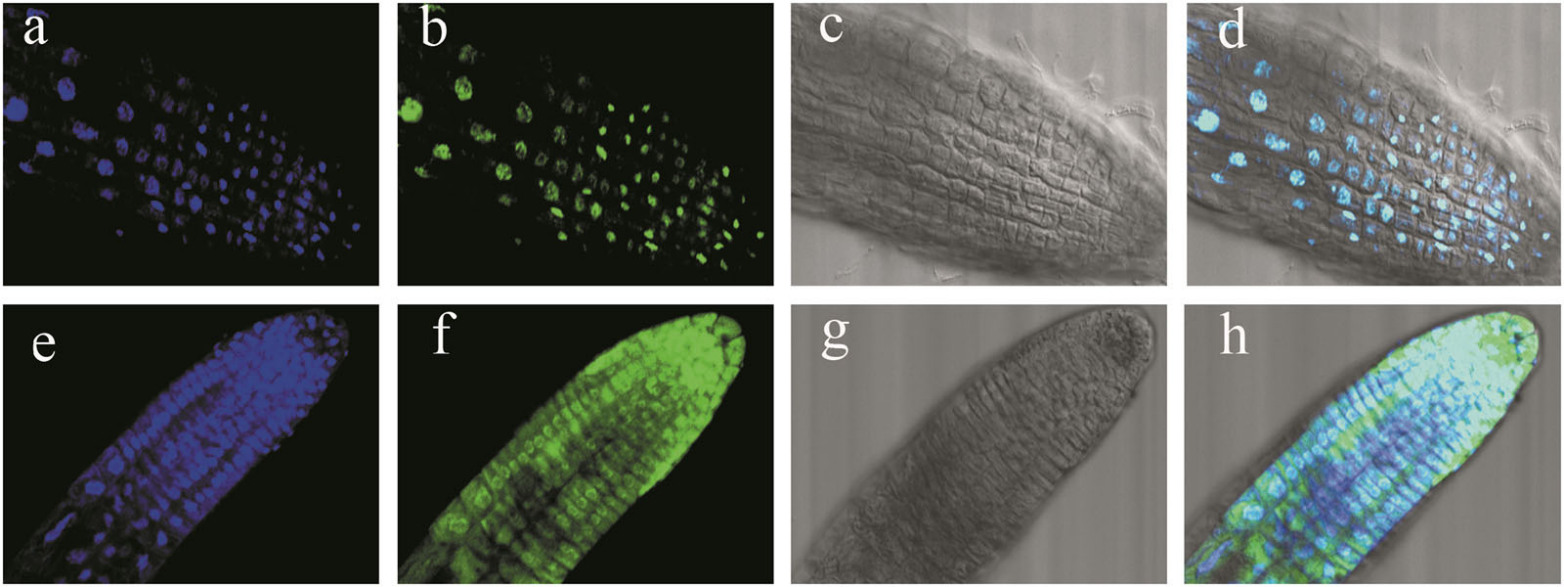
Subcellular localization of PpERF3::GFP protein in transformed Arabidopsis cells. Fluorescent signals from green fluorescent protein (GFP) were mainly detected in the nuclei of Arabidopsis cells harboring the PpERF3::GFP reporter construct. **a**, **e** DAPI; **b**, **f** GFP fluorescence; **c**, **g** bright field; **d**, **h** merged image

To examine the subcellular localization of PpERF3 in vivo, we cloned *PpERF3* and fused the full-length coding sequence without the termination codon in-frame with *GFP*. We transformed *Arabidopsis thaliana* plants with the *PpERF3::GFP* vector via *Agrobacterium*-mediated transformation and found that the fluorescent signals from the *PpERF3::GFP* fusion protein were exclusively localized to the nucleus. By contrast, fluorescent signals from the GFP control were ubiquitously distributed throughout the cell (Fig. 3).

### PpERF3 might bind to *PpNCED2/3* promoters

**Fig. 4:**
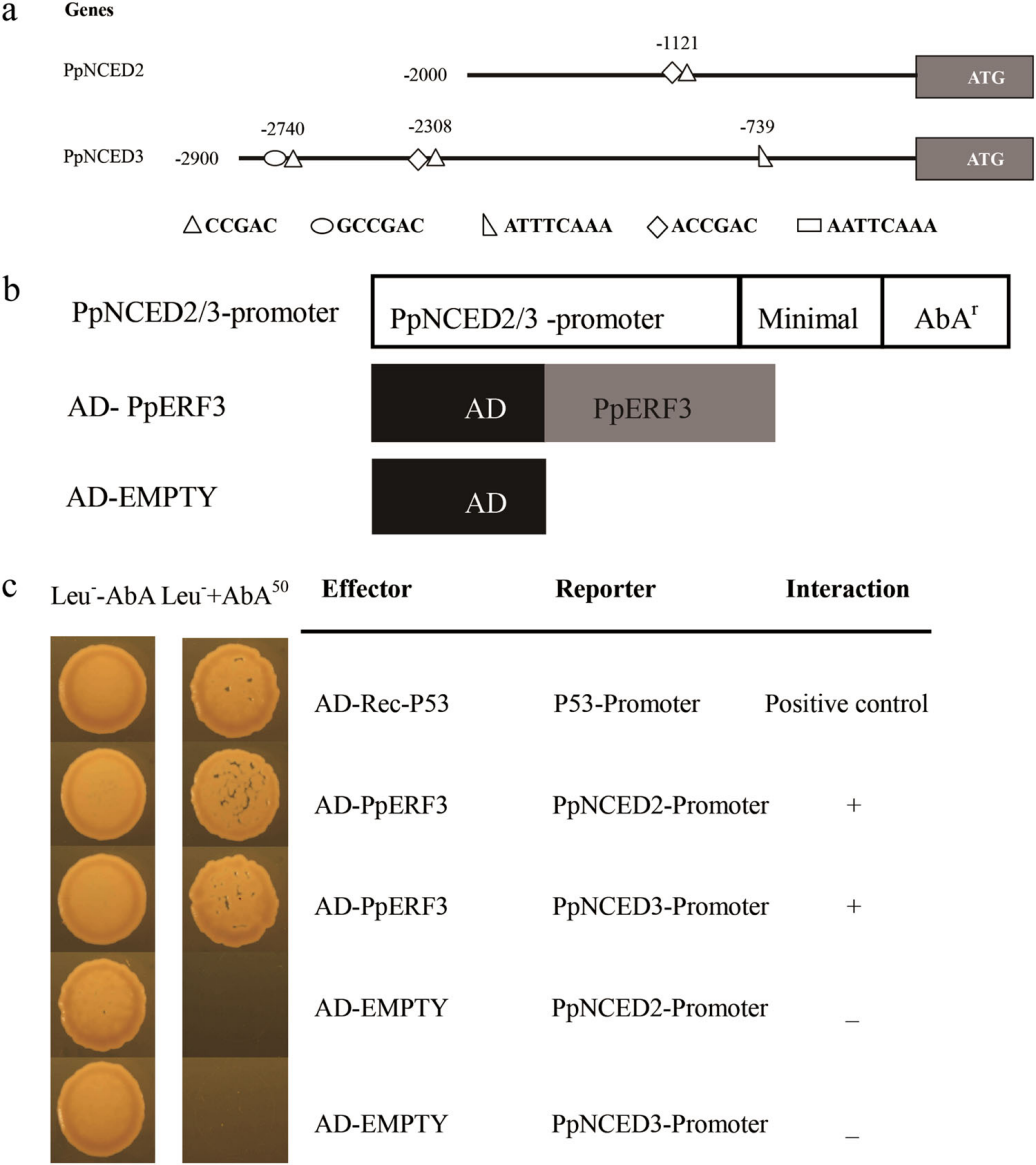
*PpNCED2/3* promoters contain ERF-binding motifs. PpERF3 might bind to the *PpNCED2/3* promoters. **a** The promoters of *PpNCED2/3* genes are represented by lines (showing promoter length); various motifs in the promoters are indicated. The exact locations of *cis*-acting elements are marked by numbers that indicate the nucleotide distance from the translation start site. **b** The CDS of *PpERF3* was cloned into the pGADT7 vector, whereas the promoters of *PpNCED2/3* were cloned into the pAbAi vector. **c** The growth status of yeasts on two different types of media after they were transformed with a combination of effector and reporter vectors is shown. Normal yeast growth on the defective medium containing the antibiotic aureobasidin A (−Leu + AbA^50^) indicates the binding of protein PpERF3 to the promoter sequences of *PpNCED2* and *PpNCED3*

Because the expression levels of *PpERF3* and *PpNCED2/3* could be enhanced by ethylene treatment and because all of these genes have important roles in fruit ripening, we investigated whether PpERF3 regulates the expression of *PpNCED2/3* during fruit ripening. First, we performed a search for *cis*-acting regulatory motif in the *PpNCED2/3* promoters. We found an ERF-binding motif ACCGAC (CCGAC) located at −1121 bp in the *PpNCED2* promoter and four ERF-binding motifs, ATTTCAAA, ACCGAC (CCGAC), and GCCGAC (CCGAC) located at −739, −2308, and −2740 bp, respectively, in the *PpNCED3* promoter (Fig. 4a).

We further performed a Y1H experiment. The CDS of *PpERF3* were cloned into the pGADT7 vector for the effector construct, and the *PpNCED2* or *PpNCED3* promoter fragment was cloned into the pAbAi vector immediately in front of the reporter gene, *AUR1-C*, for the reporter construct (Fig. 4a). When plasmids carrying cassettes constitutively expressing the *PpERF3* effectors were transformed into the Y1H reporter strains, yeast cells harboring the *PpNCED2/3* promoters grew well with aureobasidin A (50 ng mL^−1^) (Clontech, San Francisco, USA), whereas cells co-transformed with empty pGADT7 vector did not (Fig. 4b). These results suggested that PpERF3 binds to the promoters of *PpNCED2/3*.

### PpERF3 enhanced the activity of *PpNCED2/3* promoters

**Fig. 5:**
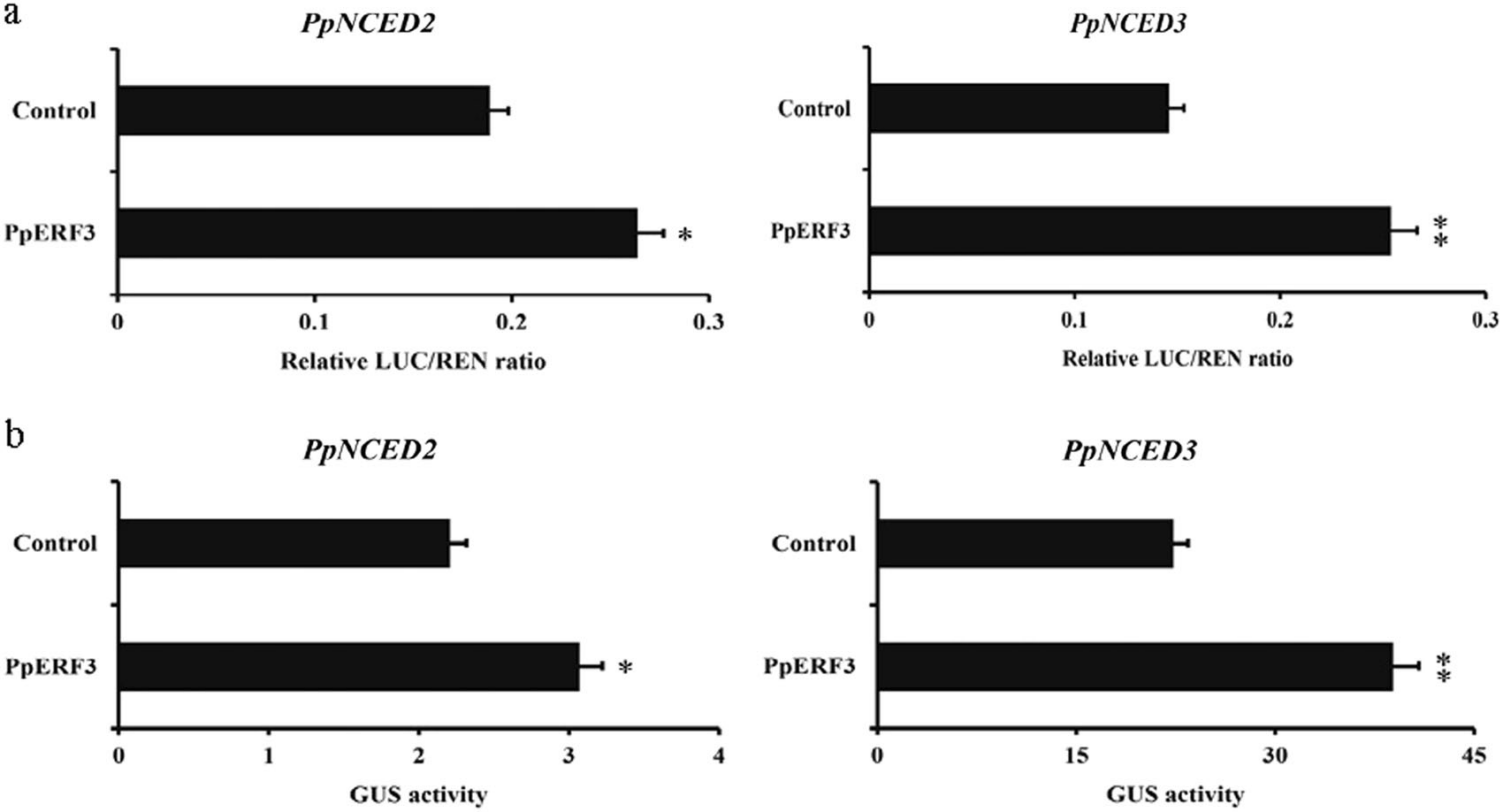
PpERF3 enhanced the activity of *PpNCED2/3* promoters in transient expression assays in tobacco leaves. **a** *Agrobacterium tumefaciens* having PpNCED2/3 or PpERF3 plasmids was infiltrated into tobacco leaves to analyze the activity enhancement of *PpNCED2/3* promoters by PpERF3. Significantly higher LUC/REN ratios were obtained with the PpERF3 effect vector than with the non-PpERF3 control vector, indicating that PpERF3 enhanced *PpNCED2/3* promoter activity. **b** The PpERF3 effector with plasmid having the *PpNCED2/3* promoters was infiltrated into tobacco leaves. The effector and empty pK2GW7 plasmids that were co-transformed into tobacco were used as controls. Values are means ±SD (n=3), * and ** represent significance at p<0.05 and p<0.01, compared to control based on t-test, respectively

To determine whether PpERF3 can enhance *PpNCED2/3* promoter activity given they can bind together, we carried out transient expression assays in tobacco leaves using dual-luciferase reporters. The assays showed that the interaction of PpERF3 with the *PpNCED2* promoter lead to a nearly 1.5-fold increase in the relative LUC/REN ratio, and the interaction of PpERF3 with the *PpNCED3* promoter lead to a nearly twofold increase in the relative LUC/REN ratio (Fig. 5a). These results suggest that PpERF3 enhances the transcription of *PpNCED2/3* by directly interacting with their promoters.

We further performed a GUS transactivation experiment by infiltrating the *35S::PpERF3* and *PpNCED2/3::GUS* plasmids into tobacco leaves. We observed significantly higher GUS activities driven by *PpNCED2/3* promoters in the assays with the *35S::PpERF3* construct than in the control assays without the *35S::PpERF3* construct (Fig. 5b). These results suggest that PpERF3 is a transcriptional activator that regulates the expression of *PpNCED2/3*.

### Transient over-expression PpERF3 in peach fruit

**Fig. 6:**
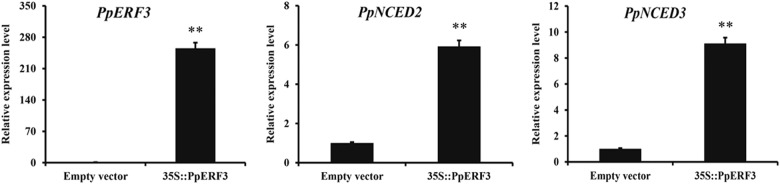
Transient over-expression of PpERF3 in peach fruit enhanced the transcript levels of *PpNCED2/3*. Transcript levels of *PpERF3, PpNCED2, PpNCED3* in peach fruit infiltrated with the empty (control) and *35* *S::PpERF3* vectors. Values are means ± SD (n=3), ** represent significance at p<0.01, compared to control based on t-test

To verify the function of PpERF3 in regulating the expression of *PpNCED2/3* in peach, ‘CN13’ fruit at stage S3 were transiently transformed using the *35S::PpERF3* construct or a control vector without *PpERF3*. The transcript of *PpERF3* was not detectable in fruit transformed with the control vector, indicating that *PpERF3* was not expressed in the fruit at stage S3. However, the *PpERF3* transcript level was significantly increased in same-stage fruit transformed with the *35S::PpERF3* construct, indicating the over-expression of *PpERF3*. The transcript levels of *PpNCED2* and *PpNCED3* were increased sixfold and ninefold, respectively, in fruit transformed with *35S::PpERF3* relative to their levels in fruit transformed with the control vector (Fig. 6). This result indicated that PpERF3 can activate the expression of both *PpNCED2* and *PpNCED3* genes in peach fruit.

## Discussion

### ABA levels and the expression profiles of *PpNCED2/3* in peach fruit

ABA has critical roles in early fruit development and ripening^9–13^. Depending on the physiological stage of the fruit, the use of exogenous ABA affects the progress of fruit ripening, leading to less or more ripe fruit^13^. When exogenous ABA was applied to peach fruit at stage S4, the expression levels of ethylene- (ACS1, ACO1), cell wall- (endo-polygalacturonase, pectin methylesterases inhibitors) and auxin-related genes were upregulated, suggesting an acceleration of ripening^13^. In our study, a marked increase in ABA concentration during peach fruit ripening was observed, suggesting that ABA accumulation at stage S4 III might accelerate the softening and ripening of peach fruit.

In fruit, the concentration of endogenous ABA is determined by the kinetic equilibrium of biosynthesis (NCED), catabolism (ABA 8′-hydroxylase), and reactivation (β-glucosidases/glucosyltransferases) genes. The spatio-temporal expression of these genes is regulated at the transcriptional level by ABA content^6^. Since the gene encoding NCED was first separated from the maize vp14 mutant^20^, it has been researched in various climacteric fruit species, including peach^13^, tomato^20^, apple^21^, and melon^22^, and non-climacteric fruits, such as orange^23^ and grape^24^. NCED is a key rate-limiting step in ABA biosynthesis^8^. In transgenic tomato fruits with *SlNCED1* silenced, cell wall degradation is inhibited due to the reduction in endogenous ABA levels. The shelf life of these fruits is longer than those of control fruits^10,11^. In strawberry, fruit ripening is affected by the downregulation of *FaNCED* genes^25^. In this study, we identified three *NCED* genes (Prupe.1G061300, Prupe.4G082000 and Prupe.4G150100) in the peach genome, and we examined the expression levels of two *PpNCED* genes in parallel with ABA content. The results suggest that these two genes may be important participants in ABA accumulation during fruit ripening. Compared with the *PpNCED2* promoter, the *PpNCED3* promoter contained many more binding sites for ERFs and other hormone-responsive proteins. This suggests that *PpNCED3* may be regulated by more complicated signal transduction pathways than is *PpNCED2*.

### PpERF3 directly actives the expression of *PpNCED2/3*

ERF transcription factors function as downstream components of ethylene signaling by regulating the expression of ethylene-responsive genes^3^. ERFs can direct regulate the expression of ripening-related genes by binding to *cis*-elements, such as DRE (CCGAC), (A/G)CC(G/C)AC, GCC box (AGCCGCC), and AA(T)TTCAAA, and affect the fruit ripening process^3,4,16,17^. There are few reports addressing whether ERFs can regulate ABA biosynthesis. Accumulating evidence supports the idea that ABA regulates ethylene biosynthesis and signaling by regulating the expression of *LeACS4* and *LeACO1*^12,13^. Kumar speculated that ethylene may affect the biosynthesis of ABA via regulating the expression of NCED^26^. In this study, phylogenetic analysis showed that PpERF3 clustered with MaERF9 (Supplementary Fig. S1a). MaERF9 from banana activates *MaACO1* promoter activity, and its expression is strongly correlated with the increase in ethylene production associated with climacteric fruit ripening^15^. We have shown here that *PpNCED2/3* promoters contain ERF-binding sites (Table 1) and that PpERF3 can bind to the *PpNCED2/3* promoter and enhance both promoter activity in tobacco leaves and the transcript levels of *PpNCED2/3* in peach fruit. In addition, *PpNCED2/3* expression is responsive to ethylene signaling. Taken together, our results suggest that ethylene may affect ABA biosynthesis via regulating the expression of *PpNCED2/3* genes by PpERF3 in peach fruit.

### Crosstalk between ABA and ethylene signaling

Fruit ripening is regulated by a complicated network of feedback and crosstalk among various phytohormone signaling pathways^26^. Increasing evidence suggests that the combined actions of auxin, gibberellins (GAs), cytokinin, ethylene, and ABA have crucial roles in the regulation of fruit ripening^26^. The present study showed that ABA content was markedly reduced by the ethylene inhibitor 1-MCP (Fig. 1c) and that the promoter activity of the ABA biosynthesis gene *PpNCED2/3* was strongly enhanced in tomato fruits by ethylene (Fig. 2). These results suggest that ethylene can regulate the biosynthesis of ABA. It is generally accepted that ethylene and ABA have important roles during fruit ripening. Fruit ABA content increased during ripening, and the initiation of fruit ripening was promoted by exogenous application of ABA but was delayed by fluridone (ABA inhibitor), indicating that ABA has a major role in fruit ripening process^18^. ABA, synergistically with ethylene, promotes softening in banana^27^. Overall, ABA and ethylene have important roles in the fruit ripening process, and the feedback regulation between ABA and ethylene biosynthesis may contribute to rapid fruit softening and a short shelf life of peach fruit.

In conclusion, *PpNCED2/3* share the same expression pattern with *PpERF3*, and the expression of these three genes can be greatly inhibited by 1-MCP treatment. PpERF3, as a transcriptional activator, regulates *PpNCED2/3* expression by binding to ERF response elements in the promoter region of *PpNCED2/3* genes, thereby increasing ABA levels. Our findings indicate that PpERF3 regulates fruit ripening by controlling the transcription of *PpNCED2/3* and provide insight into the regulatory network linking ethylene and ABA signaling.

## Materials and methods

### Fruit tissue collection

The peach cultivar ‘CN13’ grown in the research orchard of Zhengzhou Fruit Research Institute, Zhengzhou, China, was used in this study. ‘CN13’ (melting flesh; MF) fruits were harvested at 87, 90, 93, and 96 DAFB (days after full bloom). These time points corresponded to fruit development stages S3, S4 I, S4 II, and S4 III. At stage S3, the fruit is still green and at the end of the second exponential growth phase. At S4 I, the fruit is no longer inflated and does not release ethylene. At S4 II, the fruit releases low amounts of ethylene. At S4 III, the fruit release much higher levels ethylene and begins to soften rapidly^28^. Twenty fruits were collected from five different trees at each time point. Fruits collected at S4 II were treated with a solution containing 0 or 10 µl L^−1^ 1-MCP at 20 °C for 24 h. The treated fruits were then stored at 20 °C for 1, 3, and 5 days. Mesocarp tissues of the fruits were directly collected into liquid nitrogen, and stored in a −80 °C freezer for future DNA, RNA, and ABA extraction.

### Quantitation of ABA content in ‘CN13’ fruit

ABA was extracted from ‘CN13’ fruit tissues collected at the four stages using the method described by He^29^. In brief, extraction was carried out in a solution of methanol (80%, v/v) and butylated hydroxytoluene (1 mmol L^−1^) overnight at 4 °C. The extracts were centrifuged at 10,000×*g* (4 °C) for 20 min, passed through a C 18 Sep-Pak cartridge (Waters, Milford, MA) and dried in N_2_. The dried samples were dissolved in PBS buffer (0.01 mol L^−1^, pH 7.4). ABA content was determined using an enzyme-linked immunosorbent assay (ELISA), and the ELISA data were analyzed according to Weiler et al.^30^ In brief, the samples, an ABA standard, and ABA antibodies were loaded into the wells of a microtitration plate (Nunc). The plate was maintained at 37 °C for 45 min before horseradish peroxidase-labeled goat anti-rabbit immunoglobulin was added. The plate was then maintained at 37 °C for 1 h before buffered substrate (orthophenylenediamine) was applied. The enzymatic reaction was carried at 37 °C for 15 min in the dark, and then 3 mol L^−1^ H_2_SO_4_ was added to terminate the reaction. The plate was then read at 490 nm.

### RNA extraction and cDNA synthesis

RNA was extracted from the collected peach fruit tissues using a Total RNA Kit (Sangon, Shanghai, China) according to the manufacturer’s instructions. RNA samples were evaluated in 1% agarose gels to determine the levels of degradation and contamination and then analyzed using a NanoDrop ND-1000 spectrophotometer (Thermo Scientific, Wilmington, USA) to determine RNA quantity and quality. First-strand cDNA was synthesized from the RNA samples with a Reverse Transcriptase kit (Tiangen, Beijing, China). The cDNA products were diluted to 20 ng µL^−1^ for subsequent quantitative reverse-transcription PCR (qRT-PCR) analyses.

### Gene expression analysis by qRT-PCR

The relative expression levels of the *NCED* and *AP2/ERF* genes were analyzed together with an *Actin* gene as a reference^31^ using qRT-PCR. The analyses were performed with three independent biological replicates. All data were analyzed using the 2^−ΔΔCt^ method^32^. PCR primers were designed based on the sequences of the 3′-UTRs or 5′-UTRs of individual genes to ensure gene-specificity using Primer 5 software. The designed primers are listed in Supplementary Table S2. These primers produced 60 to 200 bp DNA fragments that were then cloned into pTOPO-blunt vectors and sequenced to confirm the correct amplicons of each primer pair^33^.

### Gene cloning and promoter analysis

DNA was extracted from peach leaves using a DNeasy Plant Mini Kit (Tiangen, Beijing, China). From this extracted DNA, the promoter regions of *PpNCED* genes were amplified by PCR using Phanta HS Super-Fidelity DNA Polymerase (Vazyme, Nanjing, China), cloned into the pTOPO-blunt vector (Aidlab, Beijing, China) and sequenced. DNA sequence alignments of the cloned promoters were performed using MEGA 5.0 software. The motifs or *cis*-elements in the promoter sequences were identified using the PlantCARE database (http://bioinformatics.psb.ugent.be/webtools/plantcare/html/)^34^.

### Promoter activity assay

The promoter DNA fragment (~3 kb) of *PpNCED2/3*, amplified as described above, was ligated into pCAMBIA1301 to replace the cauliflower mosaic virus (CaMV) 35S promoter. The resulting *PpNCED2/3 pro::GUS* constructs or empty vector (pCAMBIA1301) was transferred into *Agrobacterium tumefaciens* strain GV3101 by electroporation. Then, a liquid culture of *A. tumefaciens* containing the *PpNCED2/3 pro::GUS* construct or control vector was injected into Micro Tom tomato fruit at the breaker stage through the fruit stalk until the whole fruit was infiltrated. After infiltration, the transformed fruits were sliced into discs that were then immersed in a GUS-staining solution (50 mM Na-phosphate, pH 7.2, 3 mM K_3_Fe(CN)_6_, 3 mM K_4_Fe(CN)_6_, and 0.5% Triton X-100, 0.5 mg L^−1^ x-gluc) with or without 10 mM ethephon overnight at 37 °C.

### Gene characteristics and structural analysis

Neighbor-joining phylogenetic trees were constructed from the protein sequences of PpERF3 and other ERFs affecting ethylene biosynthesis and fruit ripening using the ClustalW tool in conjunction with MEGA 5.0^35^ software with 1000 bootstrap replicates. The AP2/ERF domains of these genes were aligned together with several other functionally characterized AP2/ERF-domain proteins. The deduced amino acid sequences of the *AP2/ERF* genes used to perform the phylogenetic analysis are listed in Table S3.

### Subcellular localization of PpERF3

To investigate the subcellular localization of PpERF3, a PpERF3::GFP fusion protein was constructed. The PCR product of the *PpERF3* coding sequence was cloned into the pBI121::GFP^36^ vector after digestion with the *Xba*I and *Bam*HI restriction endonucleases. The resulting fusion construct, pBI121-PpERF3::GFP, harbored *PpERF3::GFP* driven by the 35S promoter. The pBI121-PpERF3::GFP vector was transferred into *Agrobacterium tumefaciens* strain GV3101. Arabidopsis was transformed by the floral dip method as described previously^37^. 4′,6-diamidino-2-phenylindole (DAPI) was used to stain the nuclei. The subcellular localization of PpERF3-GFP was observed under a confocal laser-scanning fluorescence microscope.

### Yeast one-hybrid assay

The CDS region of *PpERF3*, amplified as described above, was cloned into the pGADT7 vector. The promoter fragment of *PpNCED2* or *PpNCED3*, amplified as described above, was ligated into the pAbAi vector. A transcription factor and promotor interaction assay was conducted using a Matchmaker™ Gold Yeast One-Hybrid Library Screening System Kit (Clontech, San Francisco, USA).

### GUS analysis

The effector construct was constructed by recombining the CDS of *PpERF3* with the flanking *att*B sites into the *attP* site of pDONR201 using GATEWAY™ BP Clonase™ Enzyme Mix (Invitrogen), followed by moving the *PpERF3* fragment from pDONR201 to the pK2GW7 destination vector containing the *attR* sites by mixing both plasmids using GATEWAY™ LR Clonase™ Enzyme Mix (Invitrogen). The *pK2GW7-PpERF3* fusion was driven by the 35S promoter. The reporter constructs were the *PpNCED2/3 pro::GUS* constructs described in the promoter activity assay. The reporter and effector constructs were transferred into *Agrobacterium tumefaciens* strain GV3101 and co-infiltrated into tobacco leaves. Each reporter-effector combination was infiltrated at least three times. Three days after infiltration, the leaves were used for GUS activity analyses. A fluorimeter (SpectraMax^®^ i3x Platform, USA) was used to measure fluorescence after the proteins were extracted from the infected tobacco leaves^38^.

### Dual-luciferase reporter assay

The promoter sequences of *PpNCED2* and *PpNCED3* were cloned into the pGreenII 0800-LUC^39^ vector, and the CDS of *PpERF3* was cloned into the pK2GW7 vector driven by the 35S promoter as an effector. A dual-luciferase assay kit was used to measure LUC and REN luciferase activity, and analysis was performed using the SpectraMax^®^ i3x Platform (USA) at 560 and 465 nm, respectively. The LUC to REN ratio was calculated. No fewer than six measurements were performed per assay.

### Transient expression in peach

The expression constructs *pK2GW7-PpERF3* were transferred into *A. tumefaciens* GV3101, and the empty vector (pK2GW7) was used as a control. Peach fruits were harvested at the S3 stage and used for transient expression assay as described by Liu at al.^40^ Briefly, infiltration was performed by submerging peach flesh cubes (1 cm thick) into *Agrobacterium* suspension and applying a vacuum (−70kPa) for 30 min. Then, the flesh cubes were sampled for RNA and subsequent expression analysis after culture on MS medium for 2 days.

## Electronic supplementary material

Phylogenetic analysis of PpERF3

all primer used in this paper

genes used to construct the phylogenetic trees

The transcriptome data of NCED
