# Supplementary material for: PpERF3 positively regulates ABA biosynthesis by activating PpNCED2/3 transcription during fruit ripening in peach
Source: Hortic Res. 2019 Feb 1;6:19. doi: 10.1038/s41438-018-0094-2 (PMC6355789; doi:10.1038/s41438-018-0094-2)
Supplement: Supplementary file 1 — Phylogenetic analysis of PpERF3 [file 41438_2018_94_MOESM1_ESM.doc]

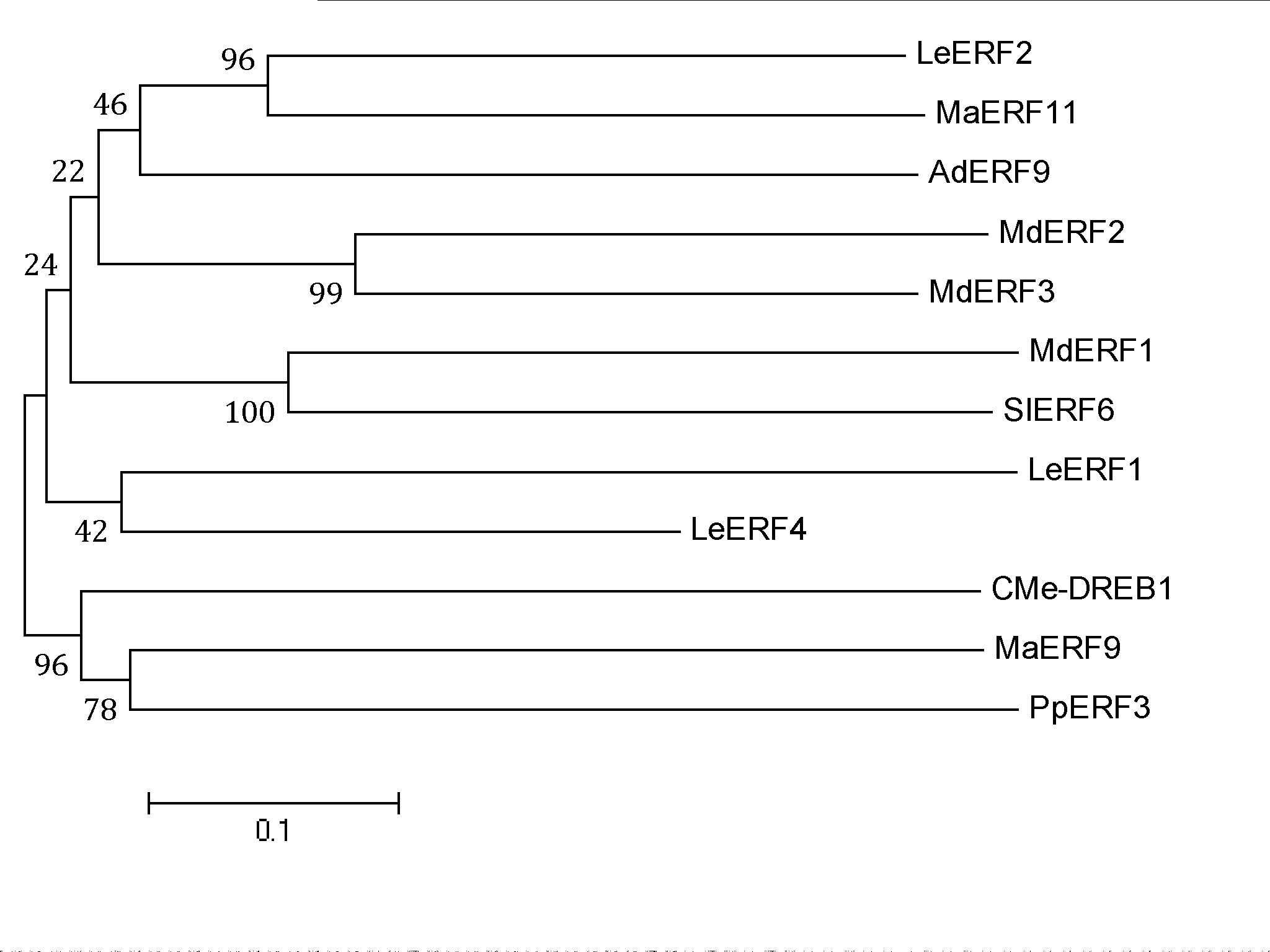

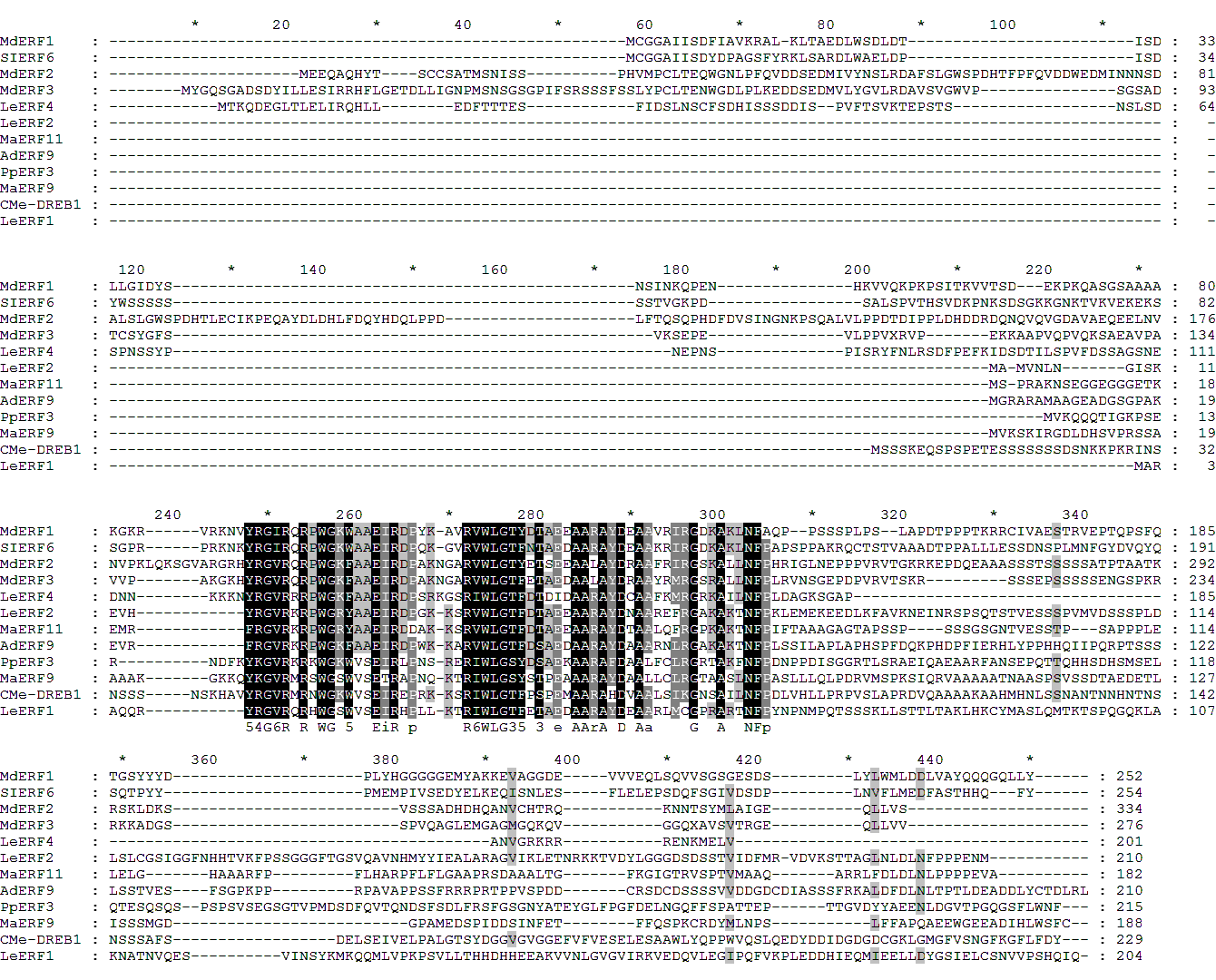


b

Fig. 1. (a) Phylogenetic analysis of PpERF3. The protein sequences used to build the phylogenetic trees include the following: LeERF1 (AY077626), LeERF2 (AY275554), LeERF4 (AY192370), SIERF6 (JN616265), AdERF9 (GQ869860), CMe-DREB1 ([NP_001306241.1](https://www.ncbi.nlm.nih.gov/protein/NP_001306241.1?report=genbank&log$=prottop&blast_rank=1&RID=AW7J27KG014)), MdERF1 ([BAF43419.1](https://www.ncbi.nlm.nih.gov/protein/BAF43419.1?report=genbank&log$=prottop&blast_rank=1&RID=AW7W3MZN01R)), MdERF2 ([NP_001280848.1](https://www.ncbi.nlm.nih.gov/protein/NP_001280848.1?report=genbank&log$=prottop&blast_rank=1&RID=AW8040Z201R)), MdERF3 ([XP_008337947.1](https://www.ncbi.nlm.nih.gov/protein/XP_008337947.1?report=genbank&log$=prottop&blast_rank=1&RID=AW82C0Z5014)), MaERF9, MaERF11. (b) Amino acid sequence alignment of PpERF3with ERF proteins from other plant species showing conserved AP2/ERF domains. Identical and similar amino acids are highlighted in black and gray, respectively. Gaps were introduced to optimize the alignment. The AP2/ERF domain is underlined.

a
