# Supplementary material for: PpERF3 positively regulates ABA biosynthesis by activating PpNCED2/3 transcription during fruit ripening in peach
Source: Hortic Res. 2019 Feb 1;6:19. doi: 10.1038/s41438-018-0094-2 (PMC6355789; doi:10.1038/s41438-018-0094-2)
Supplement: Supplementary file 2 — all primer used in this paper [file 41438_2018_94_MOESM2_ESM.docx]

Table S2 primers used in this study

| Assay | Primer sequence | | | Restriction Site |
| --- | --- | --- | --- | --- |
|  |  | Forward primer(5’-3’) | Reverse primer(5’-3’) |  |
| PCR | PpNCED2 | CCCACTTTAGCCAGGTTAGGT | GTGAGGCATTTGGGCTGTAG |  |
|  | PpNCED3 | AACGAAGGGATGTGGCTGT | TATGGTCTGCTTTTGGTTTGG |  |
| RT-qPCR | PpNCED2 | TGGCGGCGAAAATAGGAA | GCTGGTTCATGACGATCAAAA |  |
|  | PpNCED3 | TAGGGTGAGAGGTTTTGGGG | CTCACGCACCTTTTTGGC |  |
| Subcelluar  localization | PBI21-GFP-PpERF3 | gagagaacacggggactctagaATGGTGAAGCAGCAGCAGACC | cctcgcccttgctcaccatggatccGAAATTCCAGAGGAAAGACCCC | BamHI  XbaI |
| YIH | PABAi-PpNCED2 | aaatgatgaattgaaaagcttTCCAAGAGCGAAAAAGAAAAG | atacagagcacatgcctcgagGTGAGGCATTTGGGCTGTAGA | HindIII  XhoI |
|  | PABAi-PpNCED3 | aaatgatgaattgaaaagcttAACGAAGGGATGTGGCTGTTAG | atacagagcacatgcctcgagTATGGTCTGCTTTTGGTTTGGG | HindIII  XhoI |
|  | AD-PpERF3 | gccatggaggccagtgaattcATGGTGAAGCAGCAGCAGACC | atgcccacccgggtggaattcGAAATTCCAGAGGAAAGACCCC | EcoRI |
| Dual LUC  assay | PpNCED2 | ctatagggcgaattgggtaccCCCACTTTAGCCAGGTTAGGT | tatatttttggcgtcttccatGTGAGGCATTTGGGCTGTAG | NcoI  KpnI |
|  | PpNCED3 | ctatagggcgaattgggtaccAACGAAGGGATGTGGCTGT | tatatttttggcgtcttccatTATGGTCTGCTTTTGGTTTGG | NcoI  KpnI |
| GUS assay | PpNCED2 | gacctgcaggcatgcaagcttgCCCACTTTAGCCAGGTTAGGT | ttaccctcagatctaccatggtGTGAGGCATTTGGGCTGTAG | HindIII  NcoI |
|  | PpNCED3 | gacctgcaggcatgcaagcttgAACGAAGGGATGTGGCTGT | ttaccctcagatctaccatggtTATGGTCTGCTTTTGGTTTGG | HindIII  NcoI |
|  |  |  |  |  |
