# Supplementary material for: PpERF3 positively regulates ABA biosynthesis by activating PpNCED2/3 transcription during fruit ripening in peach
Source: Hortic Res. 2019 Feb 1;6:19. doi: 10.1038/s41438-018-0094-2 (PMC6355789; doi:10.1038/s41438-018-0094-2)
Supplement: Supplementary file 4 — The transcriptome data of NCED [file 41438_2018_94_MOESM4_ESM.docx]

Supplementary table 1 The transcriptome data of Prupe. 1G061300 (named as PpNCED1). Gene expression levels were measured and expressed in fragments per kilobase of exon per million fragments mapped (FPKM).

| Fruit development stage | S3 | S4 I | S4 II | S4 III |
| --- | --- | --- | --- | --- |
| PpNCED1 | 0 | 0 | 0 | 0 |
| PpNCED2 | 9.07 | 6.5 | 23.72 | 938.85 |
| PpNCED3 | 31.24 | 17.59 | 45.63 | 1862.73 |
|  |  |  |  |  |
|  | | | | |
